# Distribution of kidney diseases in Joinville, Santa Catarina: analysis of a kidney biopsy data bank between 2008 and 2019

**DOI:** 10.1590/2175-8239-JBN-2021-0165

**Published:** 2022-01-24

**Authors:** Helbert do Nascimento Lima, Luciane Monica Deboni, Viviane Calice-Silva, Giana Schlickmann, Monique Jaqueline Pereira, Leonora Zozula Blind Pope, Rodrigo Paludo de Oliveira

**Affiliations:** 1 Universidade da Região de Joinville, Faculdade de Medicina, Joinville, SC, Brasil.; 2 Clínica de Nefrologia de Joinville, SC, Brasil.; 3 Fundação Pró-Rim, Joinville, SC, Brasil.; 4 Universidade da Região de Joinville, SC, Brasil.; 5 Hospital Dona Helena, Laboratório de Anatomia Patológica, Joinville, SC, Brasil.; 6 Centro de Diagnósticos Anátomo-Patológicos, Joinville, SC, Brasil.

**Keywords:** Epidemiology, Glomerulonephritis, Pathology, Biopsy, Needle, Kidney Diseases, Epidemiologia, Glomerulonefrite, Patologia, Biópsia por Agulha, Nefropatias

## Abstract

**Introduction:**

Studies based on kidney biopsies are important for the epidemiological understanding of nephropathies.

**Objective:**

To describe the main nephropathies diagnosed through renal biopsies, and compare them with regards to gender, time, healthcare insurance and age.

**Methods:**

A population-based retrospective study that reviewed all kidney disease diagnoses obtained by biopsy of a native kidney from pathology services between 2008 and 2019 in Joinville, Brazil.

**Results:**

Of 778 biopsies performed, 44.5% were primary nephropathies and 28.5% were secondary. The highest prevalence was focal segmental glomerulosclerosis (FSGS) [18.1%], followed by tubulointerstitial nephropathy (TIN) [15.9%] and IgA nephropathy (IgAN) [9.1%]. There was a growing increase in the prevalence of TIN among elderly and uninsured patients over the period. In the multivariate analysis, among the primary glomerulopathies, males had a higher risk for the occurrence of IgAN [OR=2.02; 95% CI 1.13-3.61; p=0.018], as well as being a protective factor for the occurrence of lupus glomerulonephritis (LGN) [OR=0.20, 95% CI 0.08-0.49; p<0.001]. Advancing age and dependence on a public healthcare decreased the likelihood of having a diagnosis of LGN [OR=0.91, 95% CI 0.88-0.94, p < 0.001 and OR=0.45, CI 95 % 0.21-0.96; p = 0.036, respectively]. Patients without private healthcare insurance were more likely to have TIN [OR=1.77, 95%CI 1.16-2.70; p = 0.008].

**Conclusion:**

Sex, age and type of medical healthcare insurance may be related to the occurrence of some nephropathies. The increased risk of TIN in individuals without a private healthcare plan may be an indication of inequalities in health care.

## Resumo

**Introdução:**

Estudos com base em biópsias renais são importantes para o entendimento epidemiológico das nefropatias.

**Objetivo:**

Descrever as principais nefropatias diagnosticadas por meio de biópsias renais e comparar com relação ao gênero, período, plano de saúde e à idade.

**Métodos:**

Estudo retrospectivo de base populacional que revisou todos os diagnósticos de doenças renais obtidos por biópsia de rim nativo de serviços de patologias entre 2008 a 2019, em Joinville, Brasil.

**Resultados:**

Do total de 778 biópsias realizadas, 44,5% eram nefropatias primárias e 28,5%, secundárias. A maior prevalência foi de glomeruloesclerose segmentar e focal (GESF) [18,1%], seguida por nefropatia tubulointersticial (NTI) [15,9%] e nefropatia IgA (NIgA) [9,1%]. Houve crescente aumento da prevalência de NTI nos pacientes idosos e sem plano de saúde ao longo do período. Na análise multivariada, entre as glomerulopatias primárias, o sexo masculino apresentou maior risco para ocorrência de NIgA [OR=2,02, IC 95% 1,13-3,61; *p*=0,018], bem como foi um fator de proteção para ocorrência de glomerulonefrite lúpica (GNL) [OR=0,20, IC 95% 0,08-0,49; *p* < 0,001]. O avançar da idade e a dependência de plano público de saúde diminuíram a chance para o diagnóstico de GNL [OR=0,91, IC 95% 0,88-0,94, *p* < 0,001 e OR=0,45, IC 95% 0,21-0,96; *p* = 0,036, respectivamente]. Pacientes sem plano privado de saúde apresentaram mais chance de NTI [OR=1,77, IC 95%1,16-2,70; *p* = 0,008].

**Conclusão:**

Sexo, idade e tipo de assistência médica podem estar relacionados à ocorrência de algumas nefropatias. O maior risco de NTI em indivíduos sem plano de saúde privado pode ser um indicativo de desigualdades no cuidado à saúde.

## Introduction

According to the latest Brazilian census on chronic dialysis, around 133,000 patients were on renal replacement therapy (hemodialysis or peritoneal dialysis) in Brazil in 2018^1^. In this census, nephropathies of glomerular origin are still the third cause of dialysis for chronic kidney disease in Brazil1. In these cases, renal biopsy is an important method to confirm the etiology of this glomerular disease^2^. In Brazil, data regarding trends in glomerular and non-glomerular nephropathies confirmed by means of a renal biopsy are still limited to certain regions of the country^3^^-^ 6 or based on sampling from hospitals/nephrology centers^3^^,^ 5 ^,^ 7 ^-^ 13. In Santa Catarina, there are no population data that can contribute to a better epidemiological understanding of the prevalence of nephropathies diagnosed through renal biopsy.

Glomerulopathies can be classified as primary, when the damage is primarily renal, or as having a secondary cause, when renal damage is a consequence of another systemic disease^14^^,^ 15. Among the primary glomerulopathies, immunoglobulin A (IgA) nephropathy is the histological alteration most frequently found in many European countries^16^. On the other hand, focal segmental glomerulosclerosis (FSGS) has been the most common cause of primary nephropathy in the United States, Canada and Latin America^5^^,^ 11 ^,^ 17 ^-^ 19.

In Brazil, epidemiological data on the distribution of nephropathies diagnosed by means of biopsy mainly come from samples from large nephrology centers^7^^,^ 11. Although FSGS is the most prevalent primary nephropathy found in studies carried out in Brazil^3^^,^ 5 ^,^ 7 ^,^ 10, IgAN^4^ and membranous glomerulonephritis (MGN)^6^ have shown an increasing trend in their prevalence in some regions of the country.

Some causes of chronic kidney disease on dialysis may have an underestimated prevalence in Brazilian census studies^13^, in part because of the difficulty in accessing biopsy in many regions of the country. Thus, epidemiological studies based on temporal trends and population character can add to the knowledge of the distribution of nephropathies. The present study aimed to evaluate the prevalence of the main nephropathies diagnosed through renal biopsy in native kidneys in the last 12 years in the largest city of Santa Catarina, Joinville, and to compare their distribution by sex, age and type of medical care (private or public ).

## Methods

### Design and sampling

This is a retrospective, observational study based on a review of two databases of kidney biopsies in the city of Joinville, state of Santa Catarina, from January 2008 to December 2019. Joinville is the third largest city in southern Brazil , with an estimated population of approximately 515 thousand inhabitants, according to the Brazilian Institute of Geography and Statistics (IBGE)^20^. There are two independent pathology laboratories in the city that process all renal biopsy samples performed in the city: Pathological Anatomy Diagnostic Center (CEDAP) and Pathological Anatomy Laboratory of Dona Helena Hospital (LAP-HDH). A review of all kidney diseases diagnosed on native kidney biopsies was performed within the last 12 years of the study period, in subjects aged 18 years and older. Patients diagnosed with cancer or samples without viable renal tissue were excluded. The study was approved by the local research ethics committee (CAEE number 24429919.0.0000.5366).

### Collected variables

The variables considered were based on the data informed when the renal biopsy sample was sent to the pathology laboratory. Data on sex, age, date of biopsy, presence of private health insurance, clinical information on suspected etiology of kidney disease (primary or secondary causes) were obtained from the medical records of each laboratory.

### Histological diagnosis

The diagnosis of kidney disease was considered based on histopathological findings of optical microscopy and immunofluorescence. In addition to the histological type, nephropathies were considered primary or secondary based on the information reported by the biopsy requester regarding the presence or absence of associated systemic diseases that could be related to the kidney disease. Thus, based on histological findings and presence or absence of systemic diseases, nephropathies were divided into 4 main categories: primary glomerulopathy (PGP), secondary glomerulopathy (SGN), tubulointerstitial nephropathies (TIN) and other diagnoses (OD). PGP was defined as: IgA nephropathy (IgAN), membranous glomerulonephritis (MGN), focal segmental glomerulosclerosis (FSGS), crescentic glomerulonephritis (CrescGN), minimal lesion disease (MLD), membranoproliferative GN (MPGN), mesangioproliferative nephropathy (non-IMP MsPGN). SGP were further subdivided into lupus glomerulonephritis (LGN), dense deposit disease (amyloidosis or myeloma) and diabetic nephropathy (DN), hypertensive nephrosclerosis (AHNS) and vascular nephropathy (VascN), the latter encompassing thrombotic microangiopathy, hemolytic uremic syndrome, interstitial tubular nephritis - whether acute or chronic; and acute tubular necrosis have been encompassed as tubulointerstitial nephropathies (TIN). Other diagnoses (OD) also included all those findings not categorized in the previous descriptions.

### Statistical analyses

Categorical variables are presented by the frequency of their absolute number and percentage. Numerical variables are presented as mean and standard deviation. The chi-square test was used to compare the distributions of the main nephropathies. The distribution of the main primary and secondary glomerulopathies and tubulointerstitial nephropathies were also evaluated by age (< 45, 45-65, > 65 years), sex, type of medical care (private vs. public) and by period (2008 to 2111 , 2012 to 2015, 2016 to 2019), using the chi-square test. Univariate and multivariate analyses were performed using logistic regression to assess the association of sex, age group, type of medical care and period with the occurrence of the most prevalent histological types (FSGS, IgAN and LGN) within each specific group (PGP or SGP). Similarly, we evaluated the association of these variables concerning the occurrence of TIN in relation to all other diagnoses. The analyses were performed using the STATA version 15 software.

## Results

Of the total sample (n = 877), 27 tumor biopsies and 72 samples with no renal material available were excluded. The final sample was 778. Table 1 shows the general characteristics of the sample. The mean age of the patients who underwent renal biopsy was 45.6 ± 14.8 years; 52.6% men and 56% from the Public Healthcare System (SUS). Of the total sample, 346 (44.5%) were primary glomerulopathies and 222 (28.5%) were secondary glomerulopathies. Tubulointerstitial nephropathies were present in 124 (15.9%) patients. When considering all histological patterns, we saw the highest prevalence of FSGS (18.1%), followed by TIN (15.9%) and IgAN (9.1%).

**Table 1:** Characteristics of adult patients submitted to kidney biopsy in 2008-2019 in Joinville (n=778)

	Absolute number	Percentage or standard deviation
Gender		
Men	409	52.6
Women	369	47.4
Age, years	45.60	4.85
Private healthcare plan		
Yes	436	56.0
No	342	44.0
Period		
2008 - 2011	256	32.9
2012 - 2015	239	30.7
2016 - 2019	283	36.4
Nephropathies		
Primary glomerular disease	346	44.5
Secondary glomerular disease	222	28.5
Tubulointerstitial nephropathies	124	15.9
Other	86	11.1
Histological diagnosis		
Segmentary and focal glomerulosclerosis	141	18.1
Tubulointerstitial nephropathies	124	15.9
IgA nephropathy	71	9.1
Lupus glomerulonephritis	68	8.7
Hypertensive nephrosclerosis	60	7.7
Membranous glomerulonephritis	45	5.8
Diabetic nephropathy	42	5.4
Membranous proliferative glomerulonephritis	30	3.9
Vascular nephropathy	26	3.3
Crescent glomerulonephritis	22	2.8
Mesangioproliferative glomerulonephritis	21	2.7
Acute diffuse glomerulonephritis	18	2.3
Minimal lesion disease	16	2.1
Deposit nephropathy (amyloidosis/myeloma)	8	1.0
Other	86	11.1

When analyzing the distribution of the main etiologies and nephropathies (Table 2), there was a difference in the distribution by gender, type of medical care and period analyzed. PGP was found more frequently in men (55.4%) than in women (43.8%). SGP, on the other hand, had a higher distribution among women (38.2% vs. 26.8%). Regarding the type of medical care, a higher frequency of PGP was found in patients with a private healthcare plan, while the SGP predominated in the public network. Among the periods analyzed, there was a reduction in PGP cases over the four years analyzed, while the distribution of SGP cases remained stable over the four years considered. In relation to age group, there was a higher proportion of TIN in individuals over 65 years of age, although not significant in the analyzed sample.

**Table 2:** Differences in the distribution of nephropathies by sex, age, healthcare plan and diagnostic period (n = 778)

	Primary glomerulopathy n=346	Secondary glomerulopathy n=222	Tubulointerstitial nephropathy n=124	Total	P value
	n (%)	n (%)	n (%)	n (%)	
Sex					0.003
Women	141 (43.8%)	123 (38.2%)	58 (18.0%)	322 (100%)	
Men	205 (55.4%)	99 (26.8%)	66 (17.8%)	370 (100%)	
Age range, years					0.171
< 45	177 (53.2%)	102 (30.6%)	54 (16.2%)	333 (100%)	
45-65	139 (48.9%)	95 (33.5%)	50 (17.6%)	284 (100%)	
> 65	30 (40.0%)	25 (33.3%)	20 (26.7%)	75 (100%)	
Healthcare plan					< 0.001
Private	179 (59.3%)	85 (28.1%)	38 (12.6%)	302 (100%)	
Public	167 (42.8%)	137 (35.1%)	86 (22.1%)	390 (100%)	
Period					< 0.001
2008 a 2011	124 (57.9%)	66 (30.8%)	24 (11.2%)	214 (100%)	
2012 a 2015	115 (52.8%)	73 (33.5%)	30 (13.8%)	218 (100%)	
2016 a 2019	107 (41.2%)	83 (31.9%)	70 (26.9%)	260 (100%)	

Regarding the main diagnoses of PGP (Table 3), there was no significant difference regarding gender. Regarding the age group in the period considered, there was a significant difference in the distribution of primary etiologies and a trend in relation to the healthcare plan. There was a higher prevalence of FSGS in individuals under 65 years of age, while MPGN was more commonly found in patients over 45 years of age. The presence of RPGN and MPGN was more frequent in the older group. With regards to their healthcare insurance, there was a higher prevalence of MGN, NIgAMPGN in patients without a health insurance plan, and a higher presence of MLD and IgAN in patients with healthcare insurance. As for the analyzed periods, there was a decrease in the occurrence of NIgAMPGN in the last two periods (2012-2015 and 2016-2019) compared to the first quadrennium (2008 to 2011). Also, there was an increase in the presence of RPGN and IgAN over the four years analyzed.

**Table 3:** Differences among the main etiologies of the primary glomerular diseases by sex, age, healthcare plan and diagnostic period (n = 346)

	SFGS n = 141 (41%)	MGN n = 45 (13%)	NIgAMPGN n = 21 (6%)	MPGN n = 30 (9%)	CrescGN n = 22 (6%)	MLD n = 16 (5%)	IgAN n = 71 (20%)	Total n=346 (100%)	P value
	n (%)	n (%)	n (%)	n (%)	n (%)	n (%)	n (%)	n (%)	
Sex									0.339
Women	61 (43.3)	20 (14.2)	11 (7.8)	13 (9.2)	10 (7.1)	6 (4.3)	20 (14.2)	141 (100.0)	
Men	80 (39.0)	25 (12.2)	10 (4.9)	17 (8.3)	12 (5.9)	10 (4.9)	51 (24.9)	205 (100.0)	
Age range, years									0.037
< 45	73 (54.5)	17 (12.7)	13 (9.7)	14 (10.4)	7 (5.2)	10 (7.5)	43 (24.3)	134 (100.0)	
45-65	60 (51.3)	23 (19.7)	8 (6.8)	12 (10.3)	9 (7.7)	5 (4.3)	22 (15.8)	117 (100.0)	
> 65	8 (33.3)	5 (20.8)	0	4 (16.7)	6 (25.0)	1 (4.2)	6 (20.0)	24 (100.0)	
Healthcare plan									0.059
Private	73 (40.8)	18 (10.1)	7 (3.9)	14 (7.8)	11 (6.1)	12 (6.7)	44 (24.6)	179 (100.0)	
Public	68 (40.7)	27 (16.2)	14 (8.4)	16 (9.6)	11 (6.6)	4 (2.4)	27 (16.2)	167 (100.0)	
Period									< 0.001
2008 - 2011	49 (39.5)	14 (11.3)	19 (15.3)	13 (10.5)	4 (3.2)	4 (3.2)	21 (16.9)	124 (100.0)	
2012 - 2015	51 (44.3)	16 (13.9)	0	10 (8.7)	7 (6.1)	6 (5.2)	25 (21.7)	115 (100.0)	
2016 - 2019	41 (38.3)	15 (14.0)	2 (1.9)	7 (6.5)	11 (10.3)	6 (5.6)	25 (23.4)	107 (100.0)	

VascN: vascular nephropathy; DN: diabetic nephropathy; LGN: lupus glomerulonephritis; ASHN: arterial systemic hypertension nephropathy; ADGN: acute diffuse glomerulonephritis.

Regarding the distribution of the main causes of SGP (Table 4), there was a prevalence difference in relation to sex, age group, healthcare plan and period analyzed. Regarding gender, there was a higher prevalence of AHNS and DN in men than in women (41.1% vs. 17.6% and 30.5% vs. 10.9%, respectively), while LNG was more prevalent in women (49.6% vs. 9.5%). Regarding age group, DN and NHAS were more prevalent in older individuals (> 45 years) while LNG was more prevalent in the younger age group. Patients without a private health plan had a higher prevalence of VascN, DN and AHNS compared to individuals with a private health plan. LNG was more prevalent in patients with a private plan (45.6% vs. 23.7%). Regarding the analyzed periods, there was a decrease in VascN cases in the last two quadrennium analyzed in relation to the first quadrennium (19% to 3-4%), while there was an increase in the presence of DN in the biopsies analyzed over the quadrennium's. ADGN findings showed a decrease in prevalence in the last two quadrennium compared to the first (15.4% to 4-6%).

**Table 4:** Differences among major etiologies of secondary glomerular diseases by sex, age group, health plan, and period of diagnosis (n=214)

	VascN n = 26 (12.1%)	DN n = 42 (19.6%)	LGN n = 68 (31.8%)	HN n=60 (28,0%)	ADGN n=18 (8,4%)	Total n=214 (100%)	P value
	n (%)	n (%)	n (%)	n (%)	n (%)	n (%)	
Sex							< 0.001
Women	16 (13.4)	13 (10.9)	59 (49.6)	21 (17.6)	10 (8.4)	119(100.0)	
Men	10 (10.5)	29 (30.5)	9 (9.5)	39 (41.1)	8 (8.4)	95 (100.0)	
Age range. years							< 0.001
< 45	12 (11.9)	8 (7.9)	58 (57.4)	17 (16.8)	6 (5.9)	101 (100.0)	
45-65	11 (11.8)	28 (30.1)	10 (10.8)	33 (35.5)	11 (11.8)	93 (100.0)	
> 65	3 (15.0)	6 (30.0)	0 (0)	10 (50.0)	1 (5.0)	20 (100.0)	
Healthcare plan							0.008
Private	4 (5.1)	13 (16.5)	36 (45.6)	20 (25.3)	6 (7.6)	79 (100.0)	
Public	22 (16.3)	29 (21.5)	32 (23.7)	40 (29.6)	12 (8.9)	135 (100.0)	
Period							< 0.001
2008 - 2011	19 (29.2)	8 (12.3)	19 (29.2)	9 (13.8)	10 (15.4)	65 (100.0)	
2012 - 2015	3 (4.3)	11 (15.9)	26 (37.7)	26 (37.7)	3 (4.3)	69 (100.0)	
2016 - 2019	4 (5.0)	23 (28.7)	23 (28.7)	25 (31.3)	5 (6.3)	80 (100.0)	

VascN: vascular nephropathy; DN: diabetic nephropathy; LGN: lupus glomerulonephritis; ASHN: arterial systemic hypertension nephropathy; ADGN: acute diffuse glomerulonephritis.

In the multivariate analysis, considering sex, age, type of medical care and the period analyzed (Table 5), there was no clear association of these variables with a higher occurrence of FSGS. Regarding IgAN, men were twice as likely to occur among the primary glomerulopathies as compared to women (OR = 2.02, 95% CI 1.13 to 3.61; p = 0.018). Male gender was a protective factor for the occurrence of LNG (OR = 0.20, 95% CI 0.08-0.49; p < 0.001), as well as advancing age (OR = 0.91, CI 95 % 0.88-0.94; p < 0.001). The absence of a private plan reduced the chance of occurrence of LNG by 55% compared to patients with a private plan (OR = 0.45, 95% CI 0.21-0.96; p = 0.038). Regarding the chance of occurrence of TIN compared to other nephropathies, patients from the public healthcare system were more likely to have TIN (OR = 1.77, 95% CI 1.16-2.70; p = 0.008 ), as well as an increase of almost 3-fold the chance of occurrence of TIN in the last quadrennium compared to the first (OR=2.91, 95% CI 1.75-4.83; p < 0.001).

**Table 5:** Univariate and multivariate analysis by logistic regression concerning the occurrence of SFGS, IgAN, LGN or TIN in relation to sex, age, healthcare plan and period

	Univariate			Multivariate		
	OR	CI 95%	*p*	OR	CI 95%	*p*
SFGS						
Sex (men x women)	0.91	0.58-1.43	0.698	0.93	0.59-1.47	0.758
Age (per year of increase)	1.00	0.99-1.02	0.635	1.00	0.99-1.02	0.737
Healthcare plan (public x private)	0.84	0.54-1.31	0.443	0.88	0.56-1.39	0.595
Period						
2008 a 2011	1.00			1.00		
2012 a 2015	1.68	0.99-2.87	0.056	1.64	0.95-2.81	0.075
2016 a 2019	1.19	0.68-2.08	0.535	1.17	0.66-2.07	0.585
IgAN						
Sex (men x women)	2.00	1.13-3.54	0.017	2.02	1.13-3.61	0.018
Age (per year of increase)	0.98	0.97-1.00	0.097	0.98	0.96-1.00	0.060
Healthcare plan (public x private)	0.59	0.35-1.01	0.054	0.60	0.35-1.03	0.065
Period						
2008 a 2011	1.00			1.00		
2012 a 2015	1.36	0.71-2.60	0.348	1.42	0.73-2.76	0.301
2016 a 2019	1.49	0.78-2.86	0.224	1.56	0.79-3.07	0.197
LGN						
Sex (men x women)	0.11	0.05-0.23	< 0.001	0.20	0.08-0.49	< 0.001
Age (per year of increase)	0.90	0.87-0.93	< 0.001	0.91	0.88-0.94	< 0.001
Healthcare plan (public x private)	0.41	0.23-0.74	0.003	0.45	0.21-0.96	0.038
Period						
2008 a 2011	1.00			1.00		
2012 a 2015	1.37	0.67-2.80	0.391	2.17	0.86-5.45	0.100
2016 a 2019	0.95	0.46-1.94	0.885	1.89	0.73-4.85	0.190
TIN						
Sex (men x women)	1.06	0.70-1.60	0.797	0.94	0.63-1.39	0.936
Age (per year of increase)	1.03	1.01-1.06	0.003	1.01	0.99-1.02	0.298
Healthcare plan (public x private)	2.21	1.40-3.50	0.001	1.77	1.16-2.70	0.008
Period						
2008 a 2011	1.00			1.00		
2012 a 2015	1.08	0.58-2.02	0.797	1.39	0.79-2.47	0.256
2016 a 2019	2.83	1.66-4.81	< 0.001	2.91	1.75-4.83	< 0.001

SFGS: segmentary focal glomerulosclerosis; IgAN: IgA nephropathy; LGN: lupus glomerulonephritis; TIN: tubulointerstitial nephropathy.

## Discussion

In the present study, based on a large population sample, FSGS was the most prevalent histological type among primary glomerulopathies, and, among secondary nephropathies, LNG was the most present. A significant increase in TIN cases was found in the analyzed periods, especially in older patients without healthcare insurance and in the last four years studied. Male gender was associated with a higher occurrence of IgAN, while advancing age, male gender and the absence of a private healthcare plan decreased the chance of occurrence of LNG. On the other hand, patients without healthcare insurance had a greater chance of having TIN, with this risk being greater in the last four years analyzed.

The São Paulo registry of renal biopsies was one of the first attempts to cover a larger sample of nephropathies diagnosed through renal biopsies from 11 pathology centers in São Paulo^5^. Between 1999 and 2005, 1,844 native kidney specimens were evaluated, with approximately half of them from primary causes (54%)^5^. Another study involving 5 Brazilian regions found a clear predominance of primary glomerulopathies diagnosed through biopsies in relation to secondary ones (51% vs. 23%)^4^. Furthermore, an analysis of a large registry of renal biopsies in Germany of more than 20 years found a predominance of primary glomerulopathies^16^. Although there are differences in the way of grouping the primary and secondary causes between the studies, we found a higher predominance of primary cause glomerulopathies. In addition, there was a high prevalence of tubulointerstitial nephropathies in the studied sample compared to other studies^3^^,^ 9, and with a significant increase over the four-year periods analyzed. A higher prevalence of possible cases of tubulointerstitial nephropathies, when considered in this group of tubulointerstitial nephritis, acute tubular necrosis and other nonspecific findings, has already been pointed out in another Brazilian hospital-based study^7^. The increase in DN cases, among the causes of SGP, may also reflect a more frequent indication for renal biopsy in patients with diabetes who presented an atypical clinical picture that indicated the need for a biopsy.

Regarding the type of medical care, it is known that less than 30% of the Brazilian population has a private healthcare plan^21^, a reality that is also present in the city of the analyzed data, although in the present study just over half of the patients had a private healthcare plan. The smaller proportion of biopsies from the public healthcare system demonstrates a significant deficit of access to renal biopsy by individuals without private healthcare insurance. The higher percentage of causes of secondary nephropathies among patients without private healthcare insurance found in our results may also point to limited access to early diagnosis and treatment of systemic diseases with potential evolution to glomerular involvement and chronicity, mainly due to diabetes and hypertension. Although there is an Ordinance for care for patients with chronic kidney disease advocated by the Ministry of Health since 2014^22^, access to kidney biopsy by public services is still difficult in many regions of the country. Such difficulty in performing renal biopsies may partly explain the persistent drop in diagnoses of glomerulopathies in patients who progress to renal replacement therapy, and the growing increase in etiologies of undetermined causes (without performing a biopsy), as compared to recent years of Brazilian dialysis census^23^. This fact is suggested in a Brazilian study that re-evaluated the causes of end-stage chronic kidney disease (CKD) in patients undergoing dialysis treatment^13^. In that study, the CKD cause was critically assessed by a nephrologist, based on findings from medical records and kidney imaging/biopsy data. The highest prevalence of end-stage CKD was of undetermined cause (33%)^13^, which reinforces that access to renal biopsy in a timely manner, especially in the population without a private healthcare plan, may still be less than desired.

Regarding the most common histological types, in the present study, FSGS, IgAN and MGN were respectively the most prevalent among the primary causes, while LGN and ASHN were, respectively, among the secondary causes. In the São Paulo registry of renal biopsies, FSGS (29.7%), MGN (20.7%) and IgAN (17.8%) were the most prevalent forms of diagnosed primary glomerulopathies^5^. Among the secondary causes of glomerulopathies, lupus glomerulonephritis (66.2%) was the most found among the eleven participating centers in the registry^5^. In a retrospective study carried out at a University Hospital in Amazonas with 376 native kidney biopsies obtained over a 12-year period, FSGS (28.5%) was the most frequent diagnosis found among primary glomerulopathies, followed by membranous GN (18.8%) and minimal lesion disease (14.5%)^3^. A retrospective study carried out in two public hospitals of Pernambuco state evaluated 670 native kidney biopsies over 18 years^11^. Among the primary causes, FSGS (43%) was the most prevalent nephropathy found, followed by MGN (15%) and MLD (14%). Another study carried out in a single hospital in Paraná evaluated 131 biopsies of native kidneys between 2008 and 2012^10^. Among the primary causes, FSGS (31%) was the most diagnosed, and lupus GN was (49%) among the secondary causes^10^. When considering findings from other countries, a study involving 29 pathology laboratories on 4 continents evaluated the main findings of more than 60,000 renal biopsy specimens^24^. Diabetic glomerulosclerosis (19.1%) and FSGS (19.1%) were the main diagnoses of cases in North America. As for cases from South America (Brazil, Mexico and Colombia), LGN (38.1%) and FSGS (15.8%) were the two most prevalent. Of cases from Europe, IgA nephropathy (22.1%) and FSGS (14.9%) were the most common. And, concerning cases from Asia, IgA nephropathy (39.5%) and lupus glomerulonephritis (16.8%) were the two most common diagnoses^24^. A retrospective cohort carried out in Alberta, Canada, evaluated the distribution of nephropathies over a 30-year period, verified through renal biopsy^19^. The authors found a significant increase in the presence of diabetes-related nephropathy from 3.7% to 16% and an increase in the prevalence of tubulointerstitial nephropathy from 5.9% to 7.4% over the 30 years of observation^19^. Another study from a university hospital in Germany evaluated 1,208 kidney biopsies performed over a 24-year period (1990-2013) and found a different distribution in relation to the diagnosed nephropathies^16^. IgAN (20.3%) was the most prevalent diagnosed GN, followed by rapidly progressive GN (10.9%), NGN (8.7%), MLD (6.1%) and FSGS (6.1%)^16^.

When the variables studied in the present study were evaluated (gender, age, healthcare insurance and period analyzed) for the occurrence of the main diagnosed nephropathies, men had approximately twice the risk for the occurrence of IgAN compared to women. It is known that IgAN is more prevalent among men than women (2:1 ratio) among North American and European individuals^25^. In Brazil, based on a review of 600 IgA cases, it is estimated that the IgAN prevalence ratio among men and women is in the order of 1.24:1^26^. Advancing age and being male were protective factors for the occurrence of LGN in our sample. This finding is demonstrated in other Brazilian studies that indicate a higher occurrence of LGN among younger women^4^^,^ 7 ^,^ 10. With regards to TIN, patients from the public healthcare system had almost twice the chance of occurrence of this histological type in relation to individuals with a private healthcare plan. TIN has been frequently associated with the use of medications, mainly non-steroidal anti-inflammatory drugs (NSAIDs)^27^. In elderly individuals, TIN was the fourth most prevalent cause of acute renal failure in a Brazilian study^9^. We believe that the higher risk associated with TIN in individuals without healthcare insurance may indicate a greater use of NSAIDs, mainly for self-medication, reflecting a significant inequality in access to healthcare in this population studied. However, considering that, in the present study, cases of chronic tubulointerstitial nephritis were considered together with other acute cases affecting the tubule and interstitial region, it is not possible to rule out that chronic glomerulopathies, of difficult diagnostic definition, may have overestimated the total prevalence of TIN cases.

The present study has important limitations that need to be considered when generalizing its findings. First, this is a retrospective-based study with possible limitations in the clinical information available upon referral to the pathology laboratory. The criteria as to the primary and secondary etiology were based exclusively on the clinical impression of the requesting physician at the time of the biopsy. Thus, it is not possible to rule out an information bias with misclassification as to the primary or secondary etiology. However, this criterion has been adopted in other studies involving retrospective databases, despite its limitations^9^^,^ 16. In addition, the biopsy results were processed in two different pathology laboratories, without the concomitant review between the diagnoses issued for each service. Although both laboratories have an internal group with more than one pathologist to discuss challenging cases and a laboratory structure with optical microscopy and immunofluorescence, which follow international standardized methods, it is not possible to rule out an observation bias. On the other hand, the findings described in this paper represent the first study carried out in Santa Catarina and with a broad population-based sample. Such findings can broaden the epidemiological understanding of the region, in addition to raising new hypotheses for potential deficits in care that impact kidney diseases.

In conclusion, the present study corroborates findings already reported in other studies on trends in histopathological diagnoses of renal biopsy carried out in Brazil. Concurrently, we raise questions about the lack of access to histopathological diagnosis of kidney diseases in the public healthcare system, and possible causes of kidney disease that may be associated with this inequality of access to health care.
